# Molecular identification of phenylalanine ammonia lyase-encoding genes *EfPALs* and *EfPAL2*-interacting transcription factors in *Euryale ferox*

**DOI:** 10.3389/fpls.2023.1114345

**Published:** 2023-03-21

**Authors:** AiLian Liu, Yue Zhu, YuHao Wang, TianYu Wang, ShuPing Zhao, Kai Feng, LiangJun Li, Peng Wu

**Affiliations:** ^1^ College of Horticulture and Landscape Architecture, Yangzhou, Jiangsu, China; ^2^ Joint International Research Laboratory of Agriculture and Agri-Product Safety of Ministry of Education of China, Yangzhou University, Yangzhou, China

**Keywords:** phenylalanine ammonia-lyase (PAL), flavonoids, transcriptional regulation, HY5, ZAT11

## Abstract

Flavonoids are one of the most important secondary metabolites in plants, and phenylalanine ammonia-lyase (PAL) is the first rate-limiting enzyme for their biosynthesis. However, detailed information on the regulation of PAL in plants is still little. In this study, PAL in E. ferox was identified and functionally analyzed, and its upstream regulatory network was investigated. Through genome-wide identification, we obtained 12 putative PAL genes from E. ferox. Phylogenetic tree and synteny analysis revealed that PAL in E. ferox was expanded and mostly preserved. Subsequently, enzyme activity assays demonstrated that EfPAL1 and EfPAL2 both catalyzed the production of cinnamic acid from phenylalanine only, with EfPAL2 exhibiting a superior enzyme activity. Overexpression of EfPAL1 and EfPAL2 in Arabidopsis thaliana, respectively, both enhanced the biosynthesis of flavonoids. Furthermore, two transcription factors, EfZAT11 and EfHY5, were identified by yeast one-hybrid library assays as binding to the promoter of EfPAL2, and further luciferase (LUC) activity analysis indicated that EfZAT11 promoted the expression of EfPAL2, while EfHY5 repressed the expression of EfPAL2. These results suggested that EfZAT11 and EfHY5 positively and negatively regulate flavonoid biosynthesis, respectively. Subcellular localization revealed that EfZAT11 and EfHY5 were localized in the nucleus. Our findings clarified the key EfPAL1 and EfPAL2 of flavonoid biosynthesis in E. ferox and established the upstream regulatory network of EfPAL2, which would provide novel information for the study of flavonoid biosynthesis mechanism.

## 1 Introduction

More than 8000 flavonoids have been reported in plants, mainly divided into chalcone, flavones, dihydroflavonols, flavonols, flavanones, flavanols, anthocyanins, isoflavones and proanthocyanidins (Wen et al., 2020). Flavonoids affect the synthesis of cell walls (Saffer and Irish, 2018), mediate the phototropism of root (Tohge and Fernie, 2016), participate in the development of pollen (Lan et al., 2017), root growth (Tan et al., 2019) and color formation (Jiu et al., 2021), and also play an important role in resisting ultraviolet radiation and pathogen infection (Treutter, 2005; Wen et al., 2020). More importantly, flavonoids have the effect of promoting human health, such as antioxidant, anti-inflammatory activity and protection against cancer and diabetes (Gentile et al., 2018; Sun et al., 2021; Cui et al., 2022; Shen et al., 2022). Most of the intake of flavonoids in the human body comes from plants, and a diet rich in flavonoids has become the first choice for people (Gentile et al., 2018). In plants, the biosynthetic pathway of flavonoids is conserved. Phenylalanine was catalyzed by phenylalanine ammonia-lyase (PAL), cinnamate-4-hydroxylase (C4H), 4-coumarate CoA ligase (4CL) and chalcone synthase (CHS) to form the initial product naringenin chalcone. After that, various flavonoids were synthesized under the catalysis of chalcone isomerase, flavone synthase (FS), Isoflavone synthase (IFS), flavanone-3-hydroxylase (F3H), flavonol synthase (FLS), dihydroflavonol 4-reductase (DFR), anthocyanin synthase (ANS), anthocyanin reductase (ANR) and lecoanthocyanidins reductase (LAR) (Falcone Ferreyra et al., 2012).

Phenylalanine ammonia lyase (PAL) catalyzes the production of cinnamic acid from phenylalanine, which is the first rate-limiting step of the phenylpropane metabolic pathway and provides precursors for the biosynthesis of flavonoids (Dixon and Paiva, 1995). *PAL* is usually encoded by multiple genes, and the statistical information is listed in **Table 1**. Different subtypes of *PAL* may have different roles in different branches of the phenylpropane metabolic pathway (Huang et al., 2010). Among the 7 PAL genes identified in tea, CsPAL4 was closely associated with the accumulation of anthocyanins (Chen et al., 2022). It has been reported that the double knockout mutants of *AtPAL1* and *AtPAL2* in *Arabidopsis* have almost no synthesis of flavonoids (Rohde et al., 2004). *AmPAL1* from *Astragalus membranaceus* var. *Mongholicus* is overexpressed in tobacco, and the content of quercetin is higher than that of the wild type (Liu et al., 2006). The identification and expression analysis of *FtPAL* gene in *tartary buckwheat* showed that it played an important role in the synthesis of flavonoids (Li et al., 2010; Li et al., 2012). Identification of the function of *PAL* is necessary to further improve the biosynthetic pathway of flavonoids in plants.

**Table 1:** *PAL* gene identified in multiple species.

Species	Number of PAL	Reference
Bean (Phaseolus vulgaris L.)	3	(Liang et al., 1989)
Parsley (Petroselinum crispum)	4	(Logemann et al., 1995)
*Arabidopsis thaliana* (Linn.) Heynh.	4	(Raes et al., 2003)
Tomato (Lycopersicon esculentum L.)	26	(Chang et al., 2008)
Tobacco (Nicotiana tabacum L.)	4	(Reichert et al., 2009)
Bamboo (Bambusa oldhamii)	4	(Hsieh et al., 2011)
Cucumber (Cucumis sativus L.)	7	(Shang et al., 2012)
Watermelon (Citrullus lanatus)	12	(Dong and Shang, 2013)
*Nelumbo nucifera*	3	(Wu et al., 2014)
Maize (Zea mays L.)	7	(Zang et al., 2015)
Walnut (Juglans Regia L.)	12	(Yan et al., 2019)
Rice (Oryza sativa L.)	9	(He et al., 2020)
Grape (Vitis vinifera L.)	15	(Zhao et al., 2021)
Tea (Camellia sinensis L.)	7	(Chen et al., 2022)
Potato (Solanum tuberosum L.)	14	(Mo et al., 2022)

Many transcription factors have been reported to promote or inhibit PAL expression. In *Antirrhinum majus*, the enhanced GUS activity when MYB305 and the promoter of *PAL* were co-expressed in tobacco protoplasts indicated the activation of PAL by MYB305 (Sablowski et al., 1994). Tobacco *NtMYBAS1/2* can activate the expression of *NtPAL1*, thereby positively regulating the synthesis of phenylpropanoids in sporophyte (Yang et al., 2001). Three MYB family transcription factors in tartary buckwheat, *FtMYB13*, *FtMYB14* and *FtMYB15* can directly inhibit the expression of *FtPAL* (Zhang et al., 2018). In addition, *OsMYB30* can directly up-regulate the expression of *OsPAL6* and *OsPAL8* in rice (He et al., 2020). In addition, ZAT11, a transcription factor belonging to the zinc finger family, has previously been reported to negatively regulate nickel ion tolerance in *Arabidopsi*s (Liu et al., 2014). ELONGATED HYPOCOTYL 5 (HY5), a member of the basic leucinezipper (bZIP) family, plays a key role in light signaling pathways (Chen et al., 2013). HY5 can directly bind to the promoter of *CHS* to promote the accumulation of anthocyanins (Shin et al., 2007). Recent studies have revealed that *HY5* can promote fruit maturation (Wang et al., 2021). However, it has never been reported that these two transcription factors regulate *PAL*.

*Euryale ferox*, belonging to the *Nymphaeaceae* and genus *Euryale*, is a characteristic aquatic vegetable and has become recognized as a health food (Wu et al., 2022b). It has the effect of tonifying kidney, tonifying spleen (Song et al., 2011; Sun et al., 2014). The seed kernel is the main edible part, which is rich in 129 kinds of flavonoid compounds (Wu et al., 2021). Previously, transcriptome, proteome and metabolome analysis of seed kernel from *E. ferox* at different developmental stages revealed that *PAL* was probably the key gene for flavonoid biosynthesis (Wu et al., 2021; Wu et al., 2022a). Therefore, it is meaningful to study the functions and regulatory networks of *PAL*. In this study, *PALs* in *E. ferox* were identified at the genome-wide level and analyzed by bioinformatics for molecular characteristics, conserved motifs, gene structures and phylogenetic relationships. The potentially functional EfPAL1 and EfPAL2 were validated through *in vitro* enzymatic activity assays and overexpression in *Arabidopsis thaliana*. Subsequently, EfZAT11 and EfHY5 were confirmed to regulate *EfPAL2* expression using yeast one hybrid (Y1H) and dual-luciferase assay. Together, this study not only provides an in-depth understanding of the function of *EfPAL1* and *EfPAL2* in flavonoid biosynthesis in *E. ferox*, but also investigates the regulatory network of *EfPAL2*. This will provide new insights into the study of flavonoid biosynthesis in plants.

## 2 Materials and methods

### 2.1 Plant materials

The cultivar of *E. ferox* for the experiment was ‘ZHSQ’, which was planted in the aquatic vegetable experimental field (plot size: 18.9m^2^, water source: tap water) in Yangzhou University and grown under natural conditions (Yangzhou, China, 2019). Seed kernels were collected from 10 days after flowering (DAF10) to 40 days after flowering (DAF40). *Arabidopsis thaliana* (Columbia) and tobacco (*Nicotiana benthamiana*) seeds were preserved for our laboratory and grown in a culture chamber (at 25 ± 2°C, under 12 h of light and 12 h of darkness, Yangzhou, China) for subsequent experiments.

### 2.2 Genome-wide identification of *PALs* genes in *E. ferox*

The amino acid sequences of four PALs (AT2G37040, AT3G53260, AT3G10340, AT5G04230) from *Arabidopsis thaliana* (https://www.arabidopsis.org/) were blast in the protein database of *E. ferox* by Tbtools software (Chen et al., 2020). After that, the hidden Markov model (PF00221, http://pfam.xfam.org/) of PAL domain was also searched in the protein database of gorgonians. 12 candidate *PAL* genes were obtained by combining the results of both searches. Finally, they were further confirmed by CD-search in the NCBI database (https://www.ncbi.nlm.nih.gov/Structure/cdd/wrpsb.cgi). Molecular weight, and isoelectric point were predicted by the ExPASy tool (https://www.expasy.org/).

### 2.3 Chromosomal localization and conserved motifs, gene structure analysis of 12 candidate *EfPALs*

Conserved motifs were analyzed in MEME Suite (https://meme-suite.org/meme/tools/meme), and gene structure information was obtained from the GFF files of the *E. ferox* genome, and then all were visualized by Tbtools (Chen et al., 2020).

### 2.4 Phylogenetic tree and synteny analysis of *PAL*

Amino acid sequence alignment was performed by clustal W (MEGA 7 software), followed by neighbor-joining method to construct phylogenetic tree with 1000 bootstrap replicates, further perfected using EVOLVIEW (https://www.evolgenius.info/evolview/#/login). The amino acid sequences of all PALs are listed in **Table S1**. The chromosomal positions of PALs were obtained from annotation files of the *E. ferox* and *N. colorata* genome. The syntenic relationships of PALs in *E. ferox* and *N. colorata* were analyzed using the MCScanX in TbTools and visualized using Advanced Circos in Tbtools (Chen et al., 2020).

### 2.5 Quantitative real-time PCR analysis

Total RNA extraction from seed kernels at different developmental stages (DAF10-DAF40) of *E. ferox* and *Arabidopsis* leaves was performed using Plant RNA extraction kit (Takara, Dalian, China). Then, HiScript^®^ II Q RT SuperMix (Vazyme, NanJin, China) was applied to reverse transcription into cDNA. The 20 μL qRT-PCR reaction volume consisted of 10 μL 2×ChamQ SYBR qPCR Master Mix (Vazyme, Nanjing, China), 0.4 μL forward primer, 0.4 μL reverse primer, 1.0 μL cDNA template, and 8.2 μL ddH_2_O. *EfUBQ5* (ID: EF11G001150) and *AtActin2* (ID: AT3G18780) were used as the internal reference genes (Feng et al., 2018; Wu et al., 2022c). Primers were designed using Primer Premier 5.0. **Supplementary Table S2** for the gene specific primer sequences. The amplification was performed on a CFX-96 Real-time PCR system (Bio-Rad) with a real-time fluorescence quantitative PCR program: 95°C for 30s, 95°C for 10s, and 60°C for 30s, for a total of 40 cycles. The amplification efficiency of qPCR was determined by gradient dilution of the concentration of cDNA from *E. ferox* seed kernels 30 days after flowering (DAF30). Relative gene expression was calculated using 2^-ΔΔCT^ (Livak and Schmittgen, 2001). Each amplification reaction contained three biological and technical replicates.

### 2.6 Subcellular localization of *EfPAL1* and *EfPAL2* in *Arabidopsis thaliana* protoplasts

The ORF sequences of *EfPAL1* and *EfPAL2* were recombined into 16318-GFP vector with the restriction enzymes *Sal* I and *BamH* I (Takara, Dalian, China) using ClonExpress II One Step Cloning Kit (Vazyme, Nanjing, China). The leaves of *Arabidopsis thaliana* were placed in the enzymolysis solution (1.5% Cellulase R10, 0.4% Macerozyme R10, 0.4M Mannitol, 20mM KCl, 20mM MES) and then reacted for 2-3 h on a shaker (40 rpm) protected from light. After termination of the reaction by W5 solution (154mM NaCl, 125mM CaCl_2_, 5mM KCl, 2mM MES, pH=5.7), the protoplasts were resuspended by MMG solution (0.2M Mannitol, 15mM MgCl_2_, 4mM MES, pH=5.7). 10 μg of recombinant plasmids *p35S::PAL1-GFP* and *p35S::PAL2-GFP* were added to 200 μL of *Arabidopsis* protoplasts and left to stand for 5 min before adding 210 ul of PEG solution (PEG 4000, 0.2M Mannitol, 0.1M CaCl_2_) and left to stand for 30 min at 23°C. Then, 800uL W5 solution was added and mixed and centrifuged for 8min (23°C, 100g). The protoplasts were resuspended in 1 mL WI solution (0.5M Mannitol, 20mM KCl, 4mM MES, pH5.7), incubated overnight at 23°C in low light and then centrifuged to remove the supernatant. The protoplasts were suspended by adding 500uL WI solution, mixed with 5uL Dil, and incubated for 20min under dark conditions. The WI solution was washed the protoplasts three times. Finally, 100uL WI solution was added to resuspend the protoplasts, and the fluorescence signals were observed under ultra-high resolution laser confocal microscope (Leica, TCS SP8 STED, Germany). The empty vector was used as a control.

### 2.7 Subcellular localization of *EZAT11* and *EfHY5* in *Nicotiana benthamiana* leaves

A *Nicotiana benthamiana* transient expression system applied to the subcellular localization analysis of EfZAT11 and EfHY5. The empty vector pCAMBIA1300-35S-sGFP was digested with *Sac* I and *Xba* I (Takara, Dalian, China). The ORF sequences of EfZAT11 and EfHY5 were reconstituted into the 1300-GFP vector to form *p35S::ZAT11-GFP* and *p35S::HY5-GFP* (Vazyme, Nanjing, China). The recombinant plasmids *p35S::ZAT11-GFP* and *p35S::HY5-GFP* were transferred into *agroinfiltration* using the freeze-thaw method, and then their OD_600_ values were adjusted to 0.8-1.0 with the infection solution (100 mM Acetosyringone, 0.5 M MES, 10 mM MgCl_2_, pH5.6), mixed with nuclear marker 1:1 (v/v) and left for 2-3 hours. Six-week-old *N. benthamiana* leaves were injected with a syringe on the back of the leaves until the entire leaf was soaked. The fluorescent signals were observed under a microscope. The primers used above are listed in **Table S3**.

### 2.8 Molecular cloning, heterologous expression, and protein purification

The ORF sequences of *EfPAL1* and *EfPAL2* were inserted into the Pcold-TF vector using a double digestion method (*BamH*1 and *Sal*1). The recombinant plasmid was transferred into BL21 (DE3) *E. coli* and shaken at 37°C to an OD_600_ value of 0.4-0.6, then isopropyl-β-D-thiogalactopyranoside (IPTG, 0.5 mM) was added to induce protein expression at 16°C for 16 h. The cells were collected by centrifugation for 5 min (4°C, 13778g). The cells were resuspended in PBS buffer (137mM NaCl, 2.67mM KCl, 10mM Na_2_HPO_4_, 2mM KH_2_PO_4_), added lysozyme (1 mg/ml) and stood for 5 min, followed by sonication on ice for 20 min (run 4s with 8s interval). The supernatant was obtained after centrifugation (13778g, 4°C) for 10 min. The Ni-Agarose column (CWBIO, Jiangsu, China) is equilibrated with 10 mL of binding buffer (50mM Imidazole, 500mM NaCl, 20mM Tris-HCl, pH=7.9), the supernatant is put on the column, then the column is washed with 15 mL of binding buffer, and finally the target protein is eluted with 5 mL of elution buffer (500mM Imidazole, 500mM NaCl, 20mM Tris-HCl, pH=7.9). The purified target proteins were subjected to SDS-PAGE analysis, The concentration of the target protein was determined using Albumin Bovine V as the standard, according to the description of the Bradford protein Assay Kit (Beyotime, Shanghai, China). The primers used above are listed in **Table S3**.

### 2.9 Enzymatic assays of *EfPAL1* and *EfPAL2*

The total reaction system was 1 mL, consisting of 1mM L-Phe/L-Tyr, 10μg EfPAL1/EfPAL1 recombinant protein, and 100mM borate buffer (pH=9.0). After 5 min at 70°C, the reaction was terminated by adding 100 μL of 6 mM HCl. The reaction mixture was centrifuged at 13778g at 4°C for 10 min, and the supernatant was analyzed by HPLC (Agilent 1260). A C18 column (4.6×250 mm, 5 µm, Mars) was applied with 0.1% phosphoric acid in ultrapure water (v/v) as solvent A and acetonitrile solvent B. The elution gradients were as follows: 20% B at 0 min, 95% B at 25 min, and 20% B at 25.01 min. A flow rate of 1.0 ml/min and a column temperature of 25°C were applied and the chromatogram was obtained at 290 nm.

### 2.10 *EfPAL1* and *EfPAL2* were overexpressed in the *Arabidopsis thaliana*

The ORF sequences of EfPAL1 and EfPAL2 were inserted into the pCAMBIA1300-35S-sGFP vector by homologous recombination and then transferred into *A. tumefaciens* GV3101 cells. The infection solution (5% sucrose solution supplemented with silwet-77) was used to resuspend *A. tumefaciens* cells to reach an OD_600_ value of 0.8. The 5-6-week-old *Arabidopsis thaliana* (with the opened flowers and fruit pods removed) were selected and their flower buds were immersed into *agroinfiltration* infection solution for 20-30s, incubated in the dark for 24h and then cultured normally. The infection was repeated once a week later. Positive plants were screened on 1/2 MS solid medium containing hygromycin B (50 mg L^-1^) to T3 generation for subsequent experiments. The plant DNA extraction kit (Tiangen, Beijing) was used to extract DNA from 5-week-old *Arabidopsis* leaves. The extracted DNA was used as a template for PCR amplification with the target gene’s forward primer (EfPAL1-F/EfPAL2-F) and vector primer (GFP-R). The primers used above are listed in **Table S3**.

### 2.11 Determination of total flavonoid content

2g of 5-week-old *Arabidopsis* leaves were weighed, dried and ground into powder. The content of total flavonoids in 0.02 g leaves was determined by spectrophotometry according to the protocol of Plant Flavonoid Assay Kit (Suzhou Comin Biotechnology Co., Ltd., Suzhou, China).

### 2.12 PAL enzyme activity assay

0.1 g leaves of five-week-old *Arabidopsis thaliana* were ground on ice until homogenized, and the enzyme activity was determined according to the protocol of the Phenylalanine ammonialyase kit (Suzhou Comin Biotechnology Co., Ltd., Suzhou, China).

### 2.13 cDNA library construction and *Y1H* library screening

Seed kernels (10, 20, 30 and 40 DAF) (Wu et al., 2021)and leaves (diameter range 15-200 cm) (Wu et al., 2022b) of *E. ferox* were subjected to cDNA library construction (oebiotech, Shanghai, China). The promoter of *EfPAL2* (1000 bp immediately upstream of the *EfPAL2* open reading frame) was reconstituted into the pAbAi vector using the restriction enzymes *Kpn* I and *Sal* I. The recombinant plasmids were linearized using restriction endonuclease *Bstb*I. The linearized pPAL2-1000-PAbAi was transferred into YIH yeast using the Yeastmaker™ Yeast Transformation System 2 (Clontech) and grown on plates of SD/-Ura for three days. The colonies were resuspended with 0.9% NaCl, its OD_600_ value was adjusted to 0.002, and then coated on plates containing different concentrations (100-1000 ng/ml) of Aureobasidin A (AbA) and screened to the lowest AbA concentration that inhibited its auto-activation. The competent cell was produced with Y1HGold containing pPAL2-1000-PAbAi, then transferred to the library plasmid and incubated on SD/-Leu+AbA^100^ plates at 30°C for 3-5 days. Positive clones verified by PCR were sequenced and then annotated by blastx in NCBI (https://www.ncbi.nlm.nih.gov/). The primers used above are listed in **Table S3**.

### 2.14 *Y1H* assays

The ORF sequences of ZAT11 and HY5 were inserted into the pGADT7 AD vector *via* restriction enzymes *EcoR* I and *BamH* I. The two recombinant plasmids were then transferred into a competent made with Y1HGold cells containing pPAL2-1000-PAbAi and cultured in SD/-Leu+AbA^100^ medium at 30°C for 3-5 days.

### 2.15 Transient dual-luciferase detection and CCD imaging

*ProPAL2* was cloned into pGreenII 0800-LUC vector to fuse luciferase (LUC) reporter gene, EfZAT11 and EfHY5 were cloned into pGreenII 62-SK vectors, and then they were transformed into the *A. tumefaciens* GV3101 strain. *A. tumefaciens* cultures containing pGreen-ProPAL2 were mixed with those strains harboring pGreenII62-Sk-EfZAT11 or pGreenII62-Sk-EfHY5 at a ratio of 1:1 (v/v) and infiltrated into the *N. benthamiana* leaves after standing in darkness for 3 hours. Firefly LUC and Renilla LUC activity was detected with the Duo-LiteTM Luciferase Assay System after 3 days of incubation. Meanwhile, the leaves were sprayed with 100mM luciferin for CDD imaging. The primers used above are listed in **Table S3**.

### 2.16 Statistical analysis

Experimental data were analyzed using GraphPad Prism 8 software. Statistical significance were tested using multiple t tests (* P < 0.05, ** P < 0.01).

## 3 Results

### 3.1 Identification of *PALs* gene in *E. ferox* genome, and motif distribution, conserved structural domains and gene structure analysis

**Figure 1:**
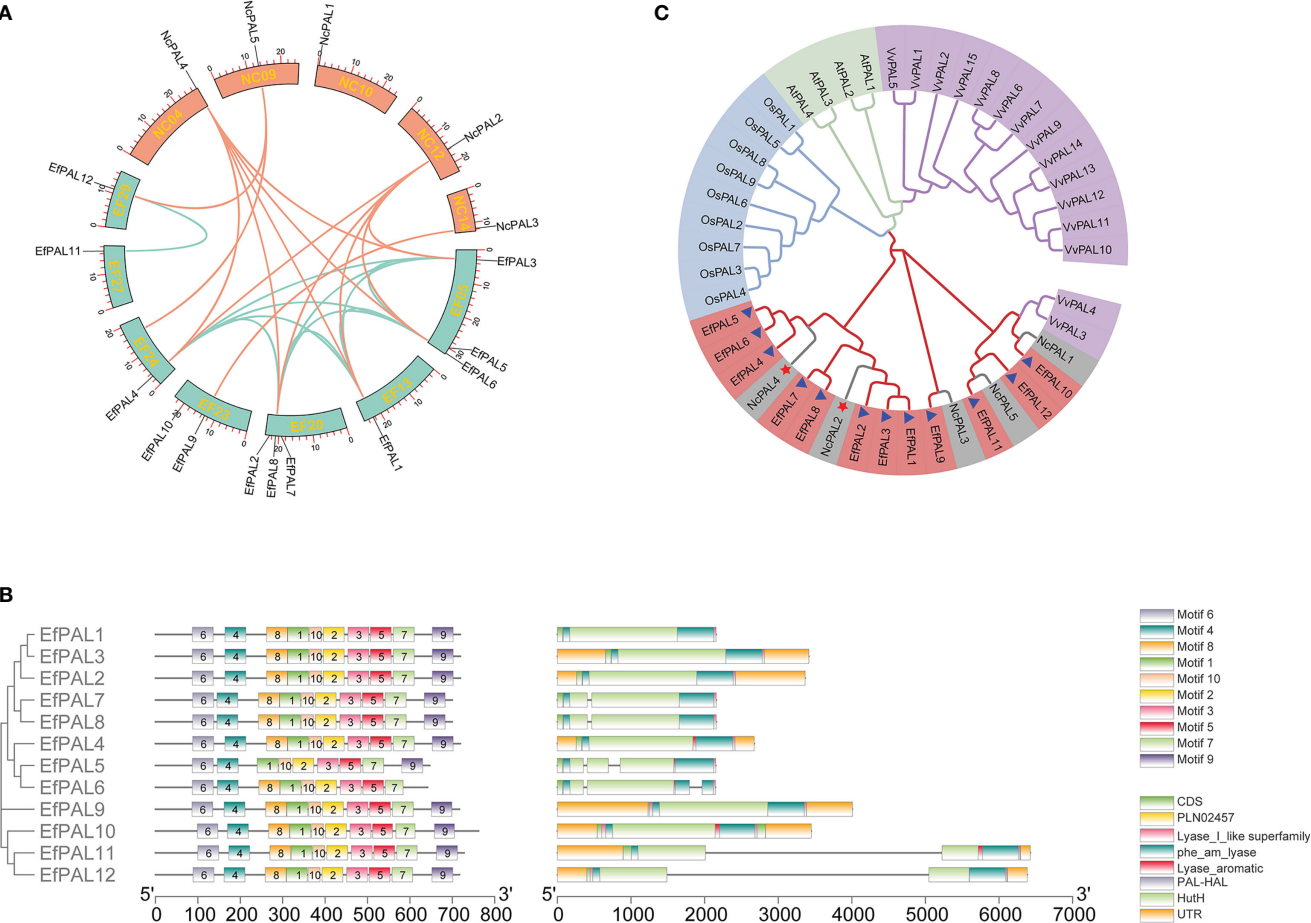
Microcollinearity analysis, motif distribution, conserved domains, gene structure, and phylogenetic analysis. **(A)** The micro-collinearity relationship of PAL in *E. ferox* and *N. colorata*. **(B)** Motif distribution, conserved domains and gene structure for the 12 putative PALs in *E. ferox*. **(C)** Phylogenetic tree of *E. ferox* PALs. Purple, green, blue, red and gray represent PALs from *Vitis vinifera*, *Arabidopsis thaliana*, *Oryza sativa*, *E. ferox*, and *Nymphaea colorata*, respectively.

Based on the hidden Markov model (HMM) and blastp function in Tbtools, 12 candidate *PAL* genes were identified in the *E. ferox* genome. The 12 putative *PAL* genes were distributed on 7 chromosomes, with *PAL1*, *PAL4*, *PAL11*, and *PAL12* located on EF13, EF24, EF27, and EF29, respectively, *PAL3*, *PAL5*, and *PAL6* were located on EF05, *PAL2*, *PAL7* and *PAL8* were located on EF20, and PAL9 and *PAL10* were located on EF23 (**Figure 1A**). The full-length coding sequences (CDS) of the 12 putative EfPALs ranged from 1926 bp to 2286 bp with amino acid numbers varying from 641aa to 761aa. There was a wide variation in their molecular weights (Mw), ranging from 69.20 KDa to 83.71 KDa, with isoelectric points (pI) between 6.08 and 6.60 (**Table 2**).

**Table 2:** Statistical analyses of sequence length, molecular weight, isoelectric point in *E. ferox.*

Gene Name	Gene ID	CDS (bp)	Amino acid residues (aa)	Molecular Weight (kDa)	Theoretical p*I*
EfPAL1	EF13G012480.2	2157	718	78.57	6.08
EfPAL2	EF20G010140.2	2157	718	78.65	6.26
EfPAL3	EF05G002400.1	2157	718	78.72	6.19
EfPAL4	EF24G001650.2	2157	718	78.47	6.14
EfPAL5	EF05G014270.2	1941	646	71.06	6.38
EfPAL6	EF05G014310.1	1926	641	69.20	6.15
EfPAL7	EF20G010120.1	2100	699	76.76	6.15
EfPAL8	EF20G010130.1	2100	699	76.66	6.26
EfPAL9	EF23G007180.1	2151	716	77.34	6.25
EfPAL10	EF23G009500.1	2286	761	83.71	6.12
EfPAL11	EF27G007800.1	2184	727	78.64	6.35
EfPAL12	EF29G005420.1	2151	716	78.07	6.60

Obviously, these 12 putative PALs with strictly conserved motifs, all of which contained motif 1, motif 2, motif 3, motif 4, motif 5, motif 6, motif 7 and motif 10. Only PAL5 had no motif 8 and PAL6 had no motif 9 (**Figure 1B**). Meanwhile, these 12 PALs all possessed five conserved structural domains, including PLN02457, phe_am_lyase, Lyase_aromatic, PAL-HAL, and HutH (**Figure 1B**). This well reflects the conservation of the PAL structural domain in *E. ferox*. In addition, gene structure analysis showed that *PAL1*, *PAL2*, *PAL3*, *PAL4*, *PAL9* and *PAL10* had no introns, *PAL7*, *PAL8*, *PAL11* and *PAL12* contained one intron, and *PAL5* and *PAL6* had two introns (**Figure 1B**). It is hypothesized that differences in gene structure may result in differences in function.

### 3.2 Phylogenetic tree and synteny analysis of *PALs*

Phylogenetic tree was constructed by using amino acid sequences of the 12 PALs in *E. ferox* with those in *Arabidopsis thaliana*, *Vitis vinifera*, *Oryza sativa* and *Nymphaea colorata*. The EfPALs are most closely related to *N. colorata*, in which NcPAL4 and NcPAL2 correspond to the three PAL genes in *E. ferox*, respectively, suggesting that these six PALs (EfPAL1-6) have been fully conserved in evolution and may have critical functions (**Figure 1C**). Besides, VvPAL3 and VvPAL4 clustered with NcPAL1 and EfPAL1. Moreover, synteny analysis was performed for PALs in *E. ferox* and *N. colorata*. As a result, there were four replication events in PALs from *E. ferox*, including 8 whole-genome duplications (WGD), 2 dispersed duplications (DSD), 1 tandem duplication (TD) and 1 proximal duplication (PD). There are 4 WGD and 1 PD presented in the PALs of *N. colorata* (**Table S4**). This suggested that whole-genome duplications (WGD) was likely to be the major driving force for *PAL* gene evolution in *E. ferox* and *N. colorata*. Meanwhile, the microcollinearity analysis demonstrated that 13 colinearity gene pairs were existed between *E. ferox* and *N. colorata*, among which NcPAL2 and NcPAL4 exhibited a one-to five relationships with EfPAL, respectively (**Figure 1A**, **Table S5**).

### 3.3 *EfPAL1* and *EfPAL2* are key candidate genes for flavonoid biosynthesis in *E. ferox*

A differential genes *PAL* (c55946) identified in the transcriptome were aligned with 12 putative *PALs* before *EfPAL1* and *EfPAL2* were selected for further study (Liu et al., 2018b). First of all, the expression patterns of *EfPAL1* and *EfPAL2* during seed kernel development (DAF10-DAF40) were analyzed by qPCR. *EfPAL1* and *EfPAL2* increased from DAF10 to DAF30 and decreased from DAF30 to DAF40, while the relative expression of *EfPAL2* was significantly higher than that of *EfPAL1* (**Figure S1**, **Table S6**). Interestingly, the expression trends of these two genes were similar to the previously measured flavonoid metabolite profiles (Wu et al., 2021), further suggesting that *EfPAL1* and *EfPAL2* are likely to be key genes for flavonoid biosynthesis in *E. ferox* seed kernels.

### 3.4 Characterization of recombinant *EfPALs* expressed in *E. coli*

**Figure 2:**
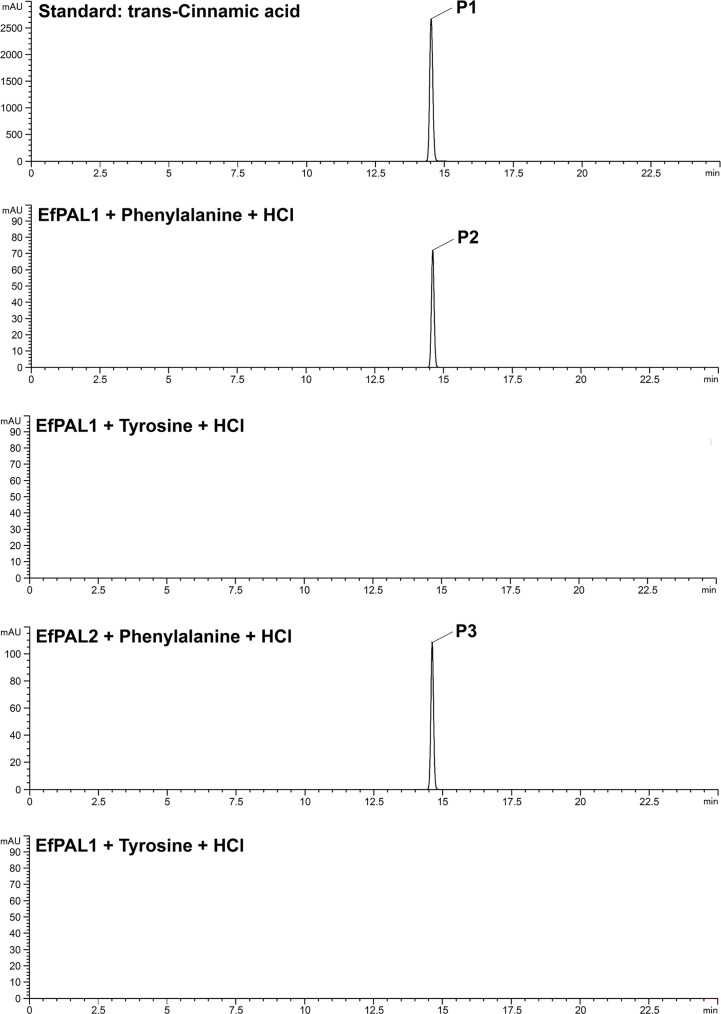
High-performance liquid chromatography analysis of the product of enzyme assays using recombinant *E. ferox* phenylalanine ammonia lyase. P1, standard cinnamic acid, P2, cinnamic acid produced by recombinant EfPAL1 enzyme reaction, P3, cinnamic acid produced by recombinant EfPAL2 enzyme reaction.

To characterize the function of EfPAL1 and EfPAL2, their CDS regions were ligated into Pcold-TF vector and transformed into *E. coli* BL21(DE3) for prokaryotic expression. The expression product with six His tags was purified by nickel column and confirmed by SDS-PAGE (**Figure S2A**). Enzyme activity analysis was performed using phenylalanine and tyrosine as substrates. With phenylalanine as the substrate, EfPAL1 and EfPAL2 could catalyze the production of trans-cinnamic acid, while with tyrosine as the substrate, the production of trans-cinnamic acid did not occur (**Figure 2**). This result revealed that EfPAL1 and EfPAL2 possessed enzymatic activity, and only for phenylalanine. Besides, the enzyme activities of purified EfPAL1 and EfPAL2 proteins were compared. As a result, the enzymatic activity of EfPAL2 was significantly higher than that of EfPAL1, which was 2.37 times higher (**Figure S2B**, **Table S7**). This suggested that EfPAL2 might play a leading role in the biosynthesis of flavonoids in *E. ferox*.

### 3.5 Activity of *EfPAL1* and *EfPAL2* in transgenic *Arabidopsis thaliana*

**Figure 3:**
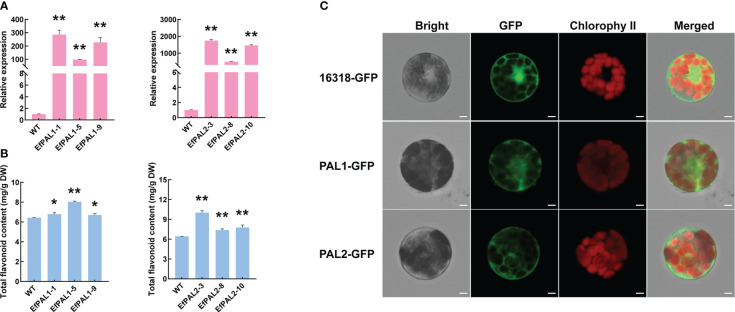
Overexpression of EfPAL1 and EfPAL2 in *Arabidopsis thaliana* and subcellular localization. **(A)** Relative expression of *PAL* in EfPAL1-OE lines and EfPAL2-OE lines. **(B)** Total flavonoid content in leaves of *Arabidopsis thaliana* of EfPAL1-OE lines and EfPAL2-OE lines. **(C)** B, Subcellular localization of EfPAL1 and EfPAL2 in the protoplasts of *Arabidopsis thaliana*, Bar=25μm. The ‘*’ or ‘**’ above the histogram indicated the statistical significance at the level of 0.05 or 0.01(p < 0.05; p < 0.01). Error bars show SD from three biological replicates.

To further demonstrate the role of EfPAL1 and EfPAL2 in flavonoid biosynthesis, we overexpressed EfPAL1 and EfPAL2 in *A. thaliana* and obtained three overexpression lines (OE-lines), respectively. Above all, target bands of 2000-3000 bp were obtained by RT-PCR amplification in three OE-lines of EfPAL1 and EfPAL2, respectively (**Figures S3A, B**). And the qPCR results showed that the expression of EfPAL1 and EfPAL2 in three OE-lines was significantly higher than that of the wild type (WT) (**Figure 3A**, **Table S8**). Subsequently, the total flavonoid content was determined and it was found that OE-lines were significantly higher than WT, indicating that EfPAL1 and EfPAL2 promote the biosynthesis of flavonoids (**Figure 3B**, **Table S8**). In addition, the PAL enzyme activity was significantly higher in the transgenic lines than in the WT (**Figure S3C**, **Table S8**). These results further suggested that EfPAL1 and EfPAL2 were the main enzymes for flavonoid biosynthesis in *E. ferox*. Then, the ORF of EfPAL1 and EfPAL2 were inserted into the 16318-GFP vector and the recombinant plasmids were transformed into *A. thaliana* protoplasts. The GFP signal was observed under laser scanning confocal microscope, with the empty vector as the control. In *A. thaliana* protoplasts transfected with *p35S::PAL1-GFP* and *p35S::PAL2-GFP*, the GFP fluorescent signal was observed mainly in the cytoplasm, while the empty vector was expressed throughout the cell (**Figure 3C**). These results indicated that EfPAL1 and EfPAL2 proteins were localized in the cytoplasm.

### 3.6 *EfZAT11* and *EfHY5* regulate the expression of *EfPAL2*

**Figure 4:**
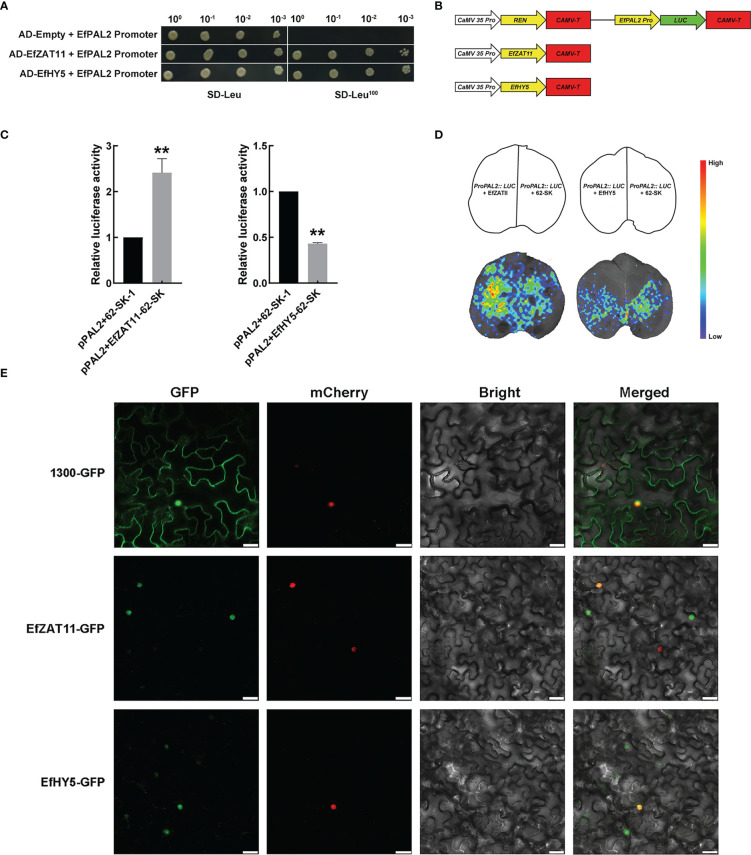
EfZAT11 and EfHY5 bind directly to the promoter of *EfPAL2* to regulate the expression of *EfPAL2* and subcellular localization. **(A)** Physical interactions between EfZAT11, EfHY5 and EfPAL2 promoter were verified by Y1H. **(B)** Schematics of transient expression vectors. REN: Rinilla luciferase **(C)** Transient dual-luciferase detections of *ProPAL2* in *N. benthamiana* leaves. **(D)** CCD image of luciferase (LUC) reporters in **(C)**. **(E)** Subcellular localization of EfZAT11 and EfHY5 in the N. benthamiana leaves, Bar=30μm. The ‘*’ or ‘**’ above the histogram indicated the statistical significance at the level of 0.05 or 0.01(p < 0.05; p < 0.01). Error bars show SD from three biological replicates.

The *EfPAL2* promoter (1000 bp) was used as a bait to screen in the *E. ferox* membrane system yeast library by yeast one-hybrid (Y1H) system, and a total of 22 colonies were obtained and sequenced (**Table S9**). Two transcription factors, ZAT11 and HY5, were identified and the YIH assay further validated the interaction of ZAT11 and HY5 with the *EfPAL2* promoter. The results showed that EfZAT11-AD+*EfPAL2* promoter and E fHY5-AD+*EfPAL2* promoter could grow on SD-Leu^100^ medium, but AD+*EfPAL2* promoter could not grow (**Figure 4A**, **Figure S4**). This indicated that EfZAT11 and EfHY5 could directly bind to the promoter of *EfPAL2* to regulate its expression. Dual luciferase assays were performed to detect the regulatory effects of EfZAT11 and EfHY5 on *EfPAL2*. When EfZAT11 was co-injected with *ProPAL2::LUC*, LUC activity was significantly increased (up-regulated 2.41-fold), while EfHY5 was co-injected with *ProPAL2::LUC*, LUC activity was significantly decreased (down-regulated 0.57-fold) (**Figures 4B, C**, **Table S10**). This suggested that EfZAT11 promoted *EfPAL2* expression and EfHY5 repressed *EfPAL2* expression. Furthermore, bioluminescence images of firefly LUC confirmed the results (**Figure 4D**).

Further, expression analysis of *EfZAT11* and *EfHY5* was performed by transcriptome and qPCR. In the transcriptome, the expression of *EfZAT11* decreased from DAF10 to DAF20 and increased from DAF20 to DAF40, which is similar to the expression trend of *PAL* (c55946) (**Figures S1**, **S5**, **Table S6**). *EfHY5* increased from DAF10 to DAF30 and decreased from DAF30 to DAF40, which is opposite to the expression trend of *c55946* (**Figures S1**, **S5**, **Table S6**). Meanwhile, qPCR analysis showed that the relative expression of *EfZAT11* increased from DAF10 to DAF20, slightly decreased from DAF20 to DAF30, and increased from DAF30 to DAF40, which is roughly similar to the expression trend of *EfPAL2* (**Figurs S1**, **S5**, **Table S6**). *EfHY5* increased from DAF10 to DAF20 and decreased from DAF20 to DAF40, which is opposite to the expression trend of *EfPAL2*. (**Figures S1**, **S5**, **Table S6**) These results indicated that EfZAT11 may promote flavonoid biosynthesis and EfHY5 inhibits flavonoid biosynthesis. Finally, the fluorescent signals of *p35S::ZAT11-GFP* and *p35S::HY5-GFP* were observed only in the nucleus and overlapped with nuclear markers, suggesting that EfZAT11 and EfHY5 are nuclear-localized transcription factors (**Figure 4E**).

## 4 Discussion

An increasing number of flavonoids in plants have been validated as functional components, and studies on their biosynthesis have been intensified (Wen et al., 2020). Although abundant flavonoids have been identified in the seed kernel of *E. ferox *(Wu et al., 2021), little research has been conducted on their biosynthetic pathways. Phenylalanine ammonia-lyase (PAL) is the first rate-limiting enzyme in phenylpropane biosynthesis, which is followed by the production of various metabolites, such as flavonoids and lignans (Zhang and Liu, 2015). The identification of *PAL* genes has occurred in many species, but information on *PAL* involved in flavonoid biosynthesis is still scarce. In our study, based on genome-wide identification, a total of 12 putative *PAL* genes were identified in *E. ferox*. They differed considerably in gene structure, with 6 *EfPAL* genes having no introns, 4 *EfPAL* having one intron and 2 *EfPALs* having two introns. In recent years, introns have been found to play unique roles in certain biological processes (Morgan et al., 2019; Parenteau et al., 2019). Thus, structural differences are likely to result in functional divergence of *PAL*. Evolutionarily, the *EfPAL1*-*EfPAL6* is completely preserved highlighting the necessity of the presence of these six genes. According to previous multi-omics analysis, *EfPAL1* and *EfPAL2* were established as key candidate genes for flavonoid biosynthesis in *E. ferox*.

The expression of the genes was closely related to the biosynthesis of the compounds (Chen et al., 2022). qPCR analysis revealed that the expression of both EfPAL1 and EfPAL2 reached the highest level at DAF30 and decreased at DAF30 to DAF40. This was in agreement with the accumulation pattern of flavonoid metabolites (Wu et al., 2021), demonstrating the potential of these two genes in flavonoid biosynthesis. Characterization of gene functions by enzymatic methods has become an important research tool and was more commonly applied in the study of flavonoids (Jiang et al., 2020; Feng et al., 2021). *In vitro* enzymatic activity assays showed that both recombinant EfPAL1 and EfPAL2 were active in catalyzing the conversion of phenylalanine to trans-cinnamic acid, while inactive against tyrosine. It has been reported that PAL in dicotyledonous plants mainly contributes to the efficient deamination of L-Phe (Zhang and Liu, 2015), and our study is in agreement with this. We overexpressed EfPAL1 and EfPAL2 in *Arabidopsis* and the total flavonoid content was significantly higher in the overexpression lines relative to the wild type. Meanwhile, the enhanced PAL enzyme activity in *Arabidopsis* also jointly confirmed that EfPAL1 and EfPAL2 are the dominant genes for flavonoid biosynthesis in gorgonians. These suggested that PAL and PAL2 in *E. ferox* are highly active for flavonoid biosynthesis and may serve as potential tool enzymes for *in vitro* production of flavonoid compounds.

HY5 is a multifunctional transcription factor involved in several processes in plants, such as cell proliferation and elongation, chlorophyll development, pigment accumulation, hormone signaling, etc (Gangappa and Botto, 2016). Notably, HY5 regulates the biosynthesis of anthocyanins reported in many crops (An et al., 2017; Liu et al., 2018a). It not only directly binds to the promoters of structural genes involved in the anthocyanin pathway and thus positively regulates anthocyanin accumulation, but also enhances anthocyanin biosynthesis by promoting regulatory factors (Shin et al., 2007; Yang et al., 2021). The latest study also revealed a new pathway for MdMPK6 to enhance MdHY5 phosphorylation and thereby promote anthocyanin accumulation (Xing et al., 2022). However, the effect of *HY5* on the biosynthesis of other flavonoids has never been reported. The *HY5* gene was obtained by performing a yeast one-hybrid screen library on the promoter of *EfPAL2*. HY5 can bind multiple cis-elements, including T/G-box, E-box, ACE-box, C-box, G-box, etc (Gangappa and Botto, 2016). The presence of a G-box on the promoter of *EfPAL2* reinforces this interaction. Interestingly, our results demonstrated that HY5 represses the expression of *EfPAL2*, suggesting that *HY5* may negatively regulate flavonoid biosynthesis in *E. ferox*. It has been reported that the stability of HY5 is positively correlated with the intensity of light (Osterlund et al., 2000), so it is speculated that the differences in gene function may be caused by the unique growth environment (underwater) of *E. ferox* seeds. Moreover, *ZAT11* is another transcription factor screened that has never before been reported for its contribution in flavonoid biosynthesis. ZAT11, a C2H2-type zinc finger protein, was another selected transcription factor that promoted primary root growth and reduced nickel tolerance in *Arabidopsis thaliana* (Liu et al., 2014). Its contribution to flavonoid biosynthesis has not been previously reported. In this study, EfZAT11 could directly bind to the promoter of *EfPAL2* and thus enhance the expression of *EfPAL2*. This suggested that EfZAT11 might positively regulate flavonoid biosynthesis in *E. ferox*. Furthermore, the expression pattern of *EfZAT11* during *E. ferox* seed kernel development was consistent with *EfPAL2*, while the opposite was true for *EfHY5*, further confirming our conclusion. In addition, both EfZAT11 and EfHY5 are localized in the nucleus, which is also consistent with the localization of most transcription factors.

## 5 Conclusions

**Figure 5:**
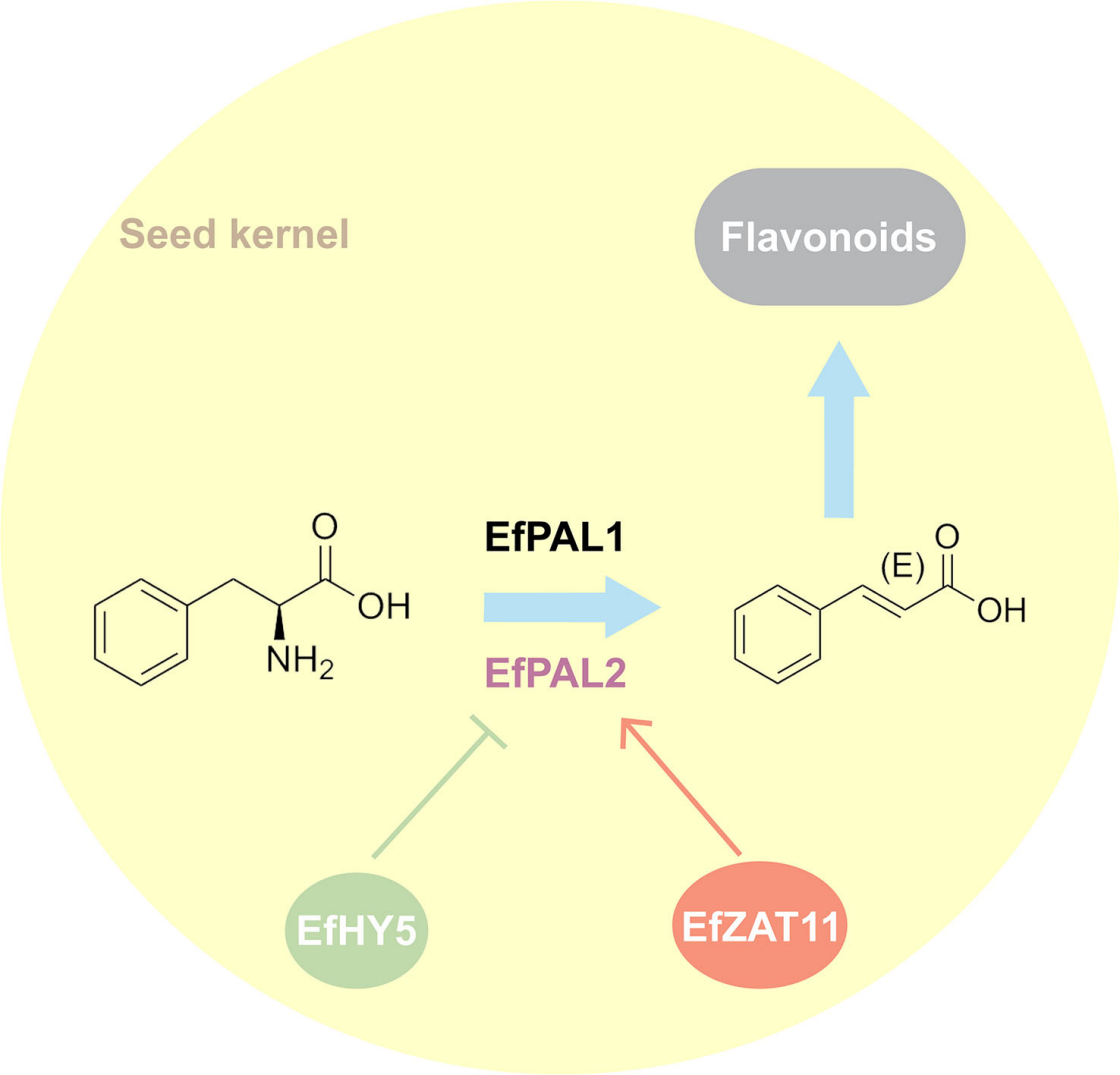
Hypothesized pattern of ZAT11 and HY5 mediating flavonoid accumulation through regulation of *PAL*.

Despite the genome-wide identification and analysis of PAL in many species, its contribution in flavonoid biosynthesis as well as its regulatory network remains not well understood. Our study identified 12 putative *PALs* from the *E. ferox* genome. Using enzyme activity assays and transgenic techniques, we confirmed that EfPAL1 and EfPAL2 contribute to the biosynthesis of flavonoids, with EfPAL2 exhibiting more significant activity. Further, two transcription factors, ZAT11 and HY5, were evidenced to bind to the promoter of EfPAL2 thereby positively or negatively regulating its expression (**Figure 5**). The present study enhances the understanding of the position of *PAL* in flavonoid biosynthesis and its upstream regulation, remedying the absence of research on this issue in plants.

## Data availability statement

The datasets presented in this study can be found in online repositories. The names of the repository/repositories and accession number(s) can be found in the article/**Supplementary Material**. All Illumina Sequencing data have been deposited in NCBI’s Sequence Read Archive (SRA) under accession number SAMN07167649 (https://www.ncbi.nlm.nih.gov/biosample/SAMN07167649/).

## Author contributions

PW and LL supervised the project. PW and AL conceived and designed the experiment. AL performed most of the experiments. AL analyzed the data and wrote the paper. YZ helped in data re-analysis. YW and TW helped with the preparation of plant materials. All authors contributed to the article and approved the submitted version.
