# Supplementary material for: Molecular identification of phenylalanine ammonia lyase-encoding genes EfPALs and EfPAL2-interacting transcription factors in Euryale ferox
Source: Front Plant Sci. 2023 Mar 21;14:1114345. doi: 10.3389/fpls.2023.1114345 (PMC10064797; doi:10.3389/fpls.2023.1114345)
Supplement: Supplementary file 1 [file DataSheet_1.docx]

**Figure S1 Expression of *EfPAL1* and *EfPAL2* during *E. ferox* seed kernel development (DAF10-DAF40) by qPCR and transcriptome together.** The ‘*’ or ‘**’ above the histogram indicated the statistical significance at the level of 0.05 or 0.01(p < 0.05; p < 0.01). Error bars show SD from three biological replicates.

**Figure S2 SDS-PAGE electropherograms and enzyme activity assays of purified recombinant EfPAL1 and EfPAL2. (**A) SDS-PAGE electropherograms of purified recombinant EfPAL1 and EfPAL2. (B) Enzyme activity assays of purified recombinant EfPAL1 and EfPAL2. The ‘*’ or ‘**’ above the histogram indicated the statistical significance at the level of 0.05 or 0.01(p < 0.05; p < 0.01). Error bars show SD from three biological replicates.

**Figure S3 Electropherograms of *EfPAL1* and *EfPAL2* amplification and determination of PAL crude enzyme activity in transgenic *Arabidopsis thaliana*. (**A) *Arabidopsis thaliana* after overexpression of EfPAL1 and EfPAL2, Bar=1cm. (B) Amplification electropherograms of *EfPAL1* and *EfPAL2*. (C) Determination of PAL crude enzyme activity in transgenic *Arabidopsis thaliana*. The ‘*’ or ‘**’ above the histogram indicated the statistical significance at the level of 0.05 or 0.01(p < 0.05; p < 0.01). Error bars show SD from three biological replicates.

**Figure S4 The validation of *EfPAL2* promoter autoactivation.**

**Figure S5 The expression levels of *EfZAT11* and *EfHY5* during *E. ferox* seed kernel development (DAF10-DAF40) was analyzed by transcriptome and qPCR.**
