# Supplementary figures and images for: Molecular identification of phenylalanine ammonia lyase-encoding genes EfPALs and EfPAL2-interacting transcription factors in Euryale ferox
Source: Front Plant Sci. 2023 Mar 21;14:1114345. doi: 10.3389/fpls.2023.1114345 (PMC10064797; doi:10.3389/fpls.2023.1114345)

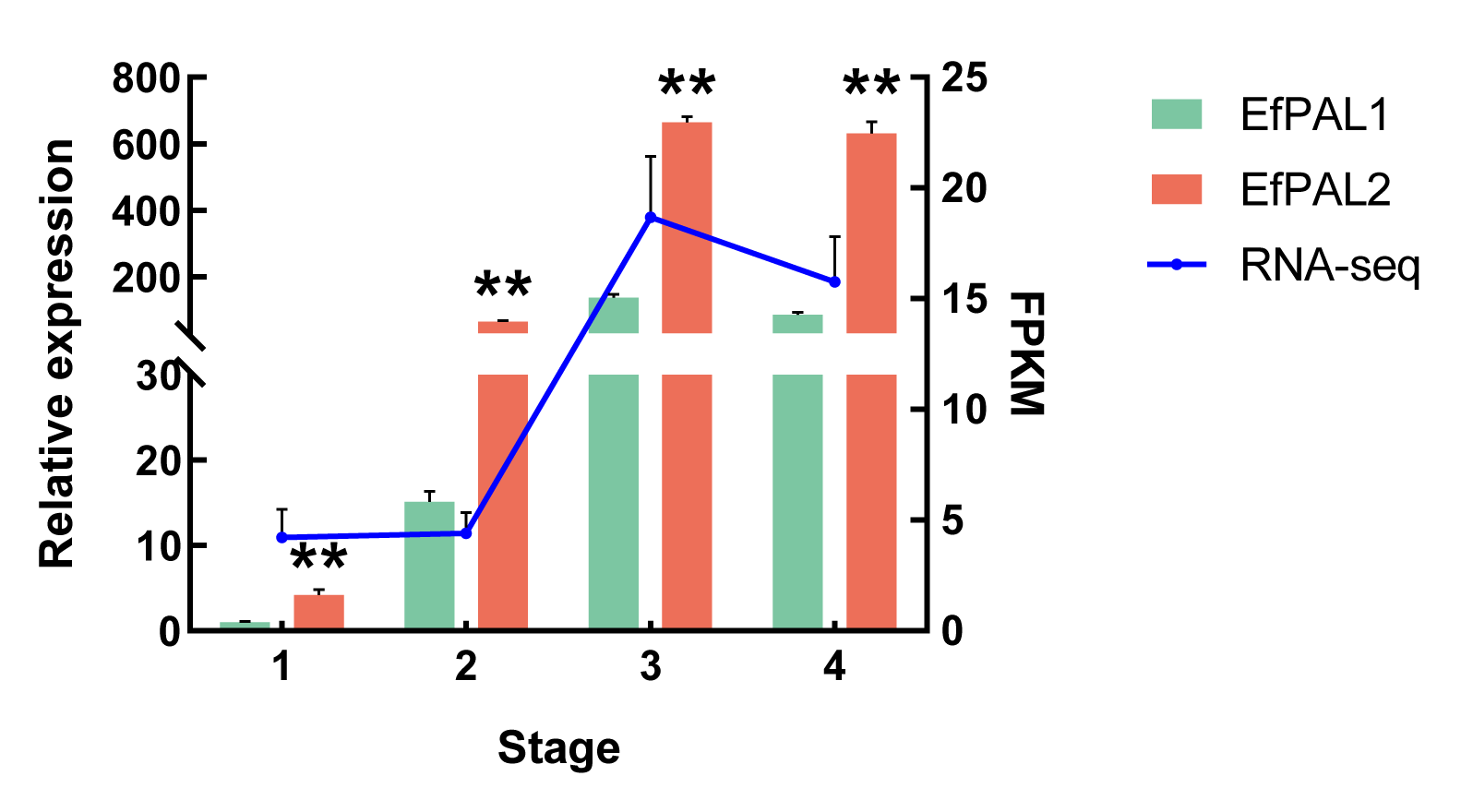

Supplement: Supplementary file 2 [file Image_1.tif]

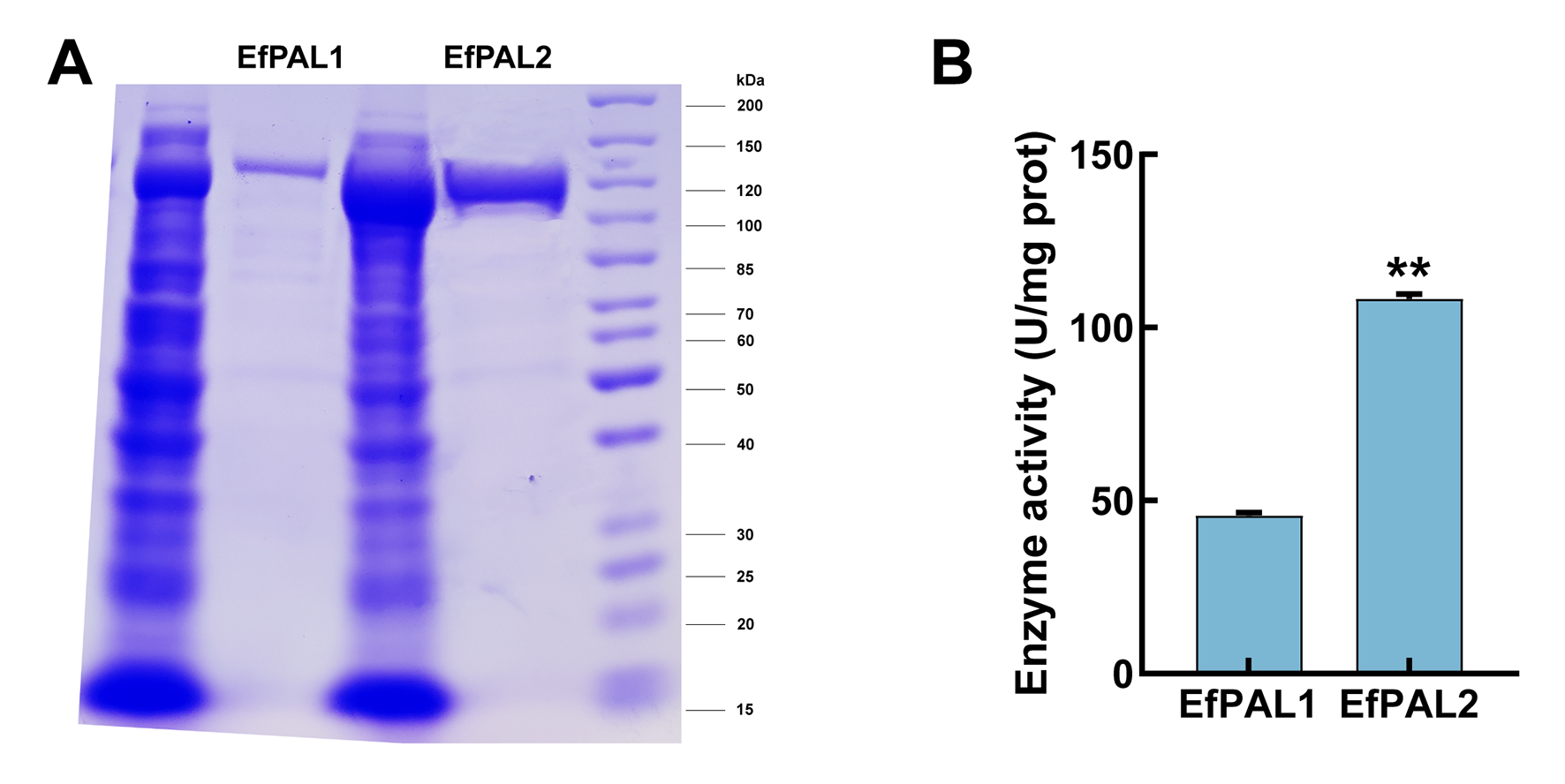

Supplement: Supplementary file 3 [file Image_2.tif]

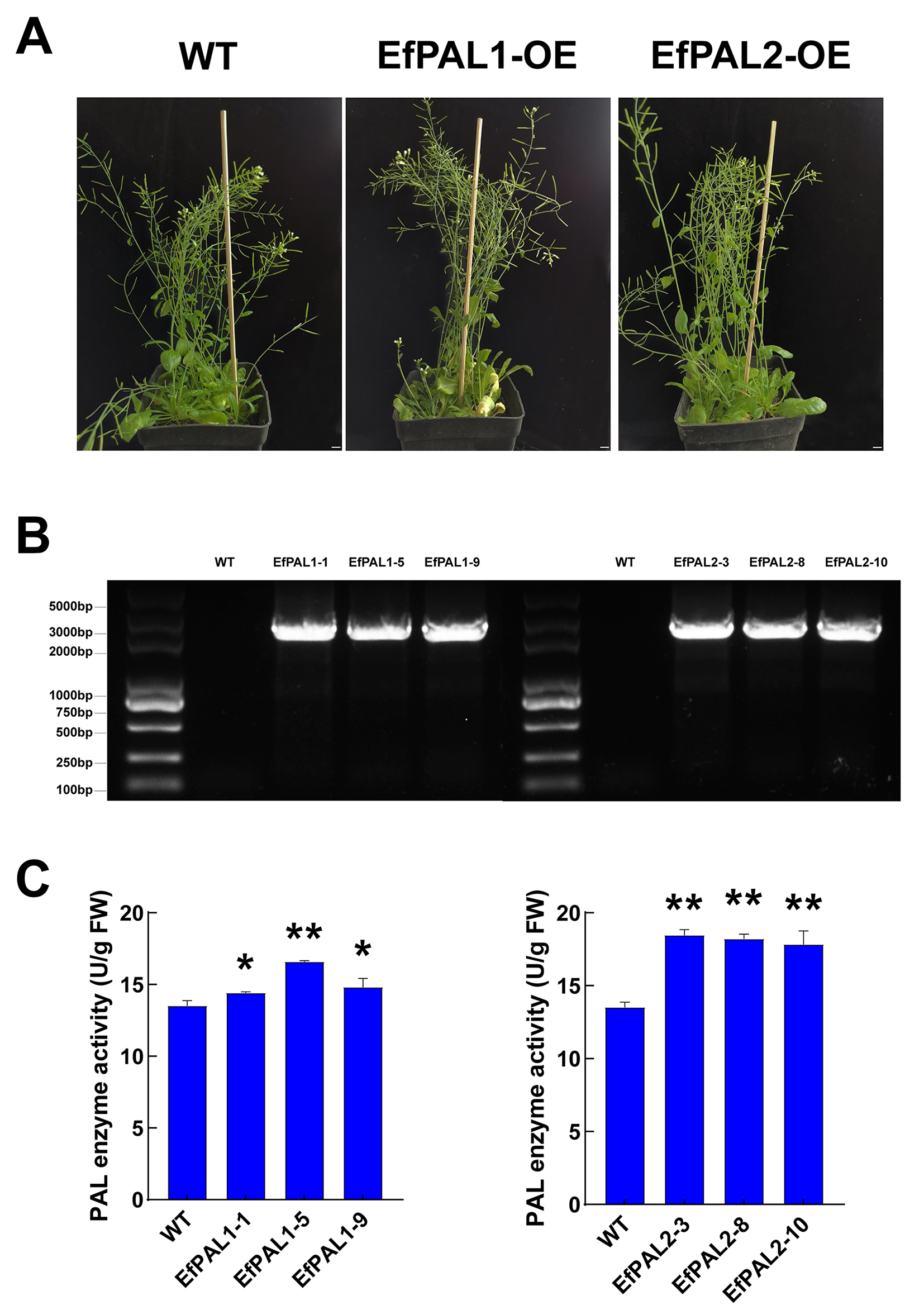

Supplement: Supplementary file 4 [file Image_3.tif]

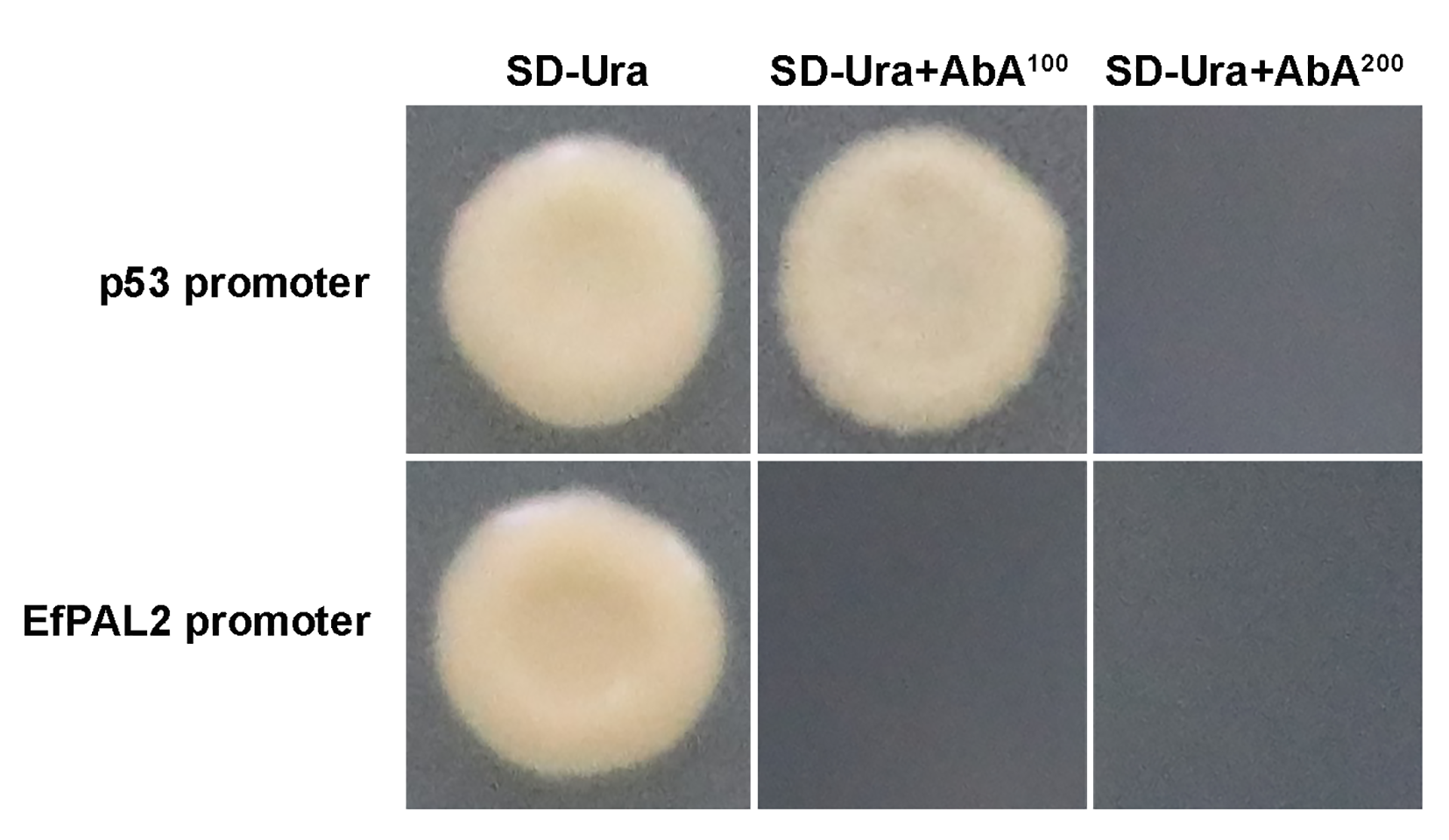

Supplement: Supplementary file 5 [file Image_4.tif]

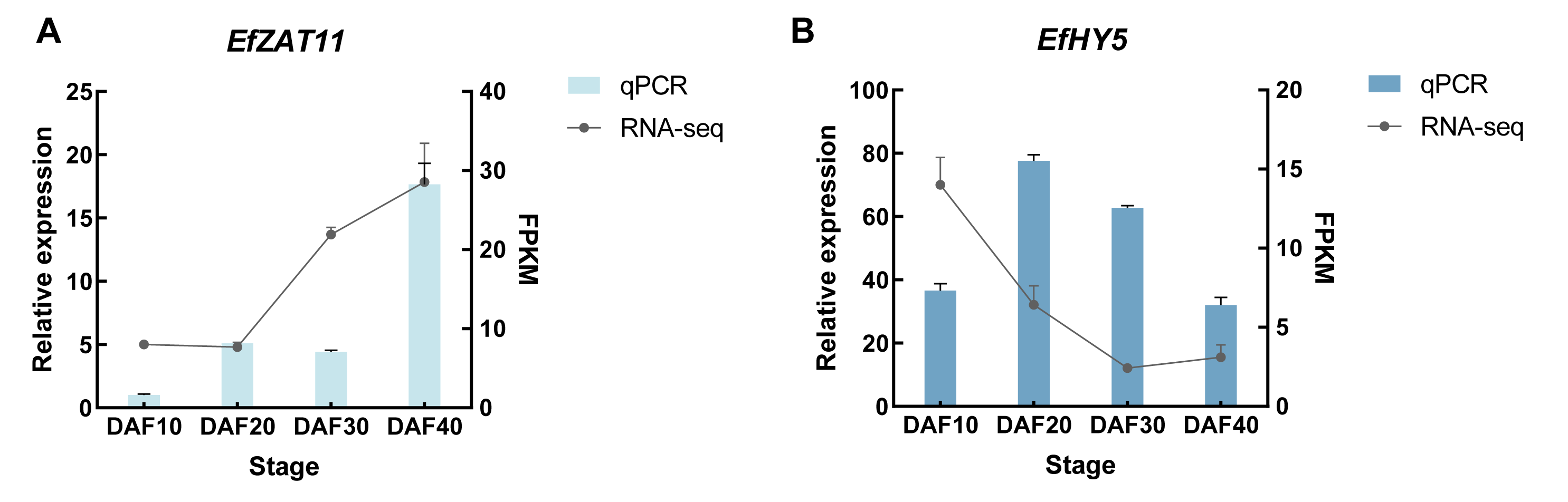

Supplement: Supplementary file 6 [file Image_5.tif]

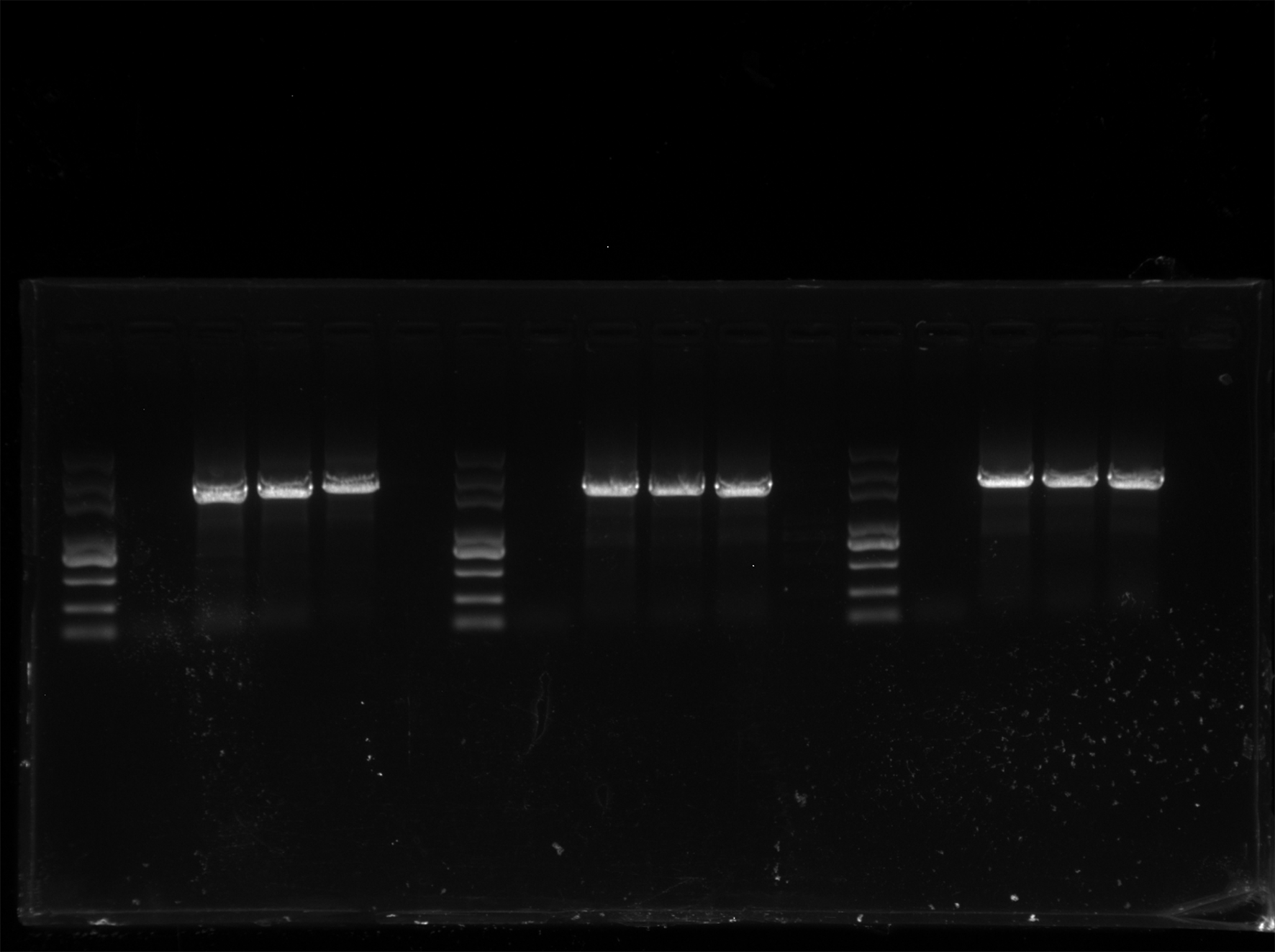

Supplement: Supplementary file 7 [file Image_6.tif]

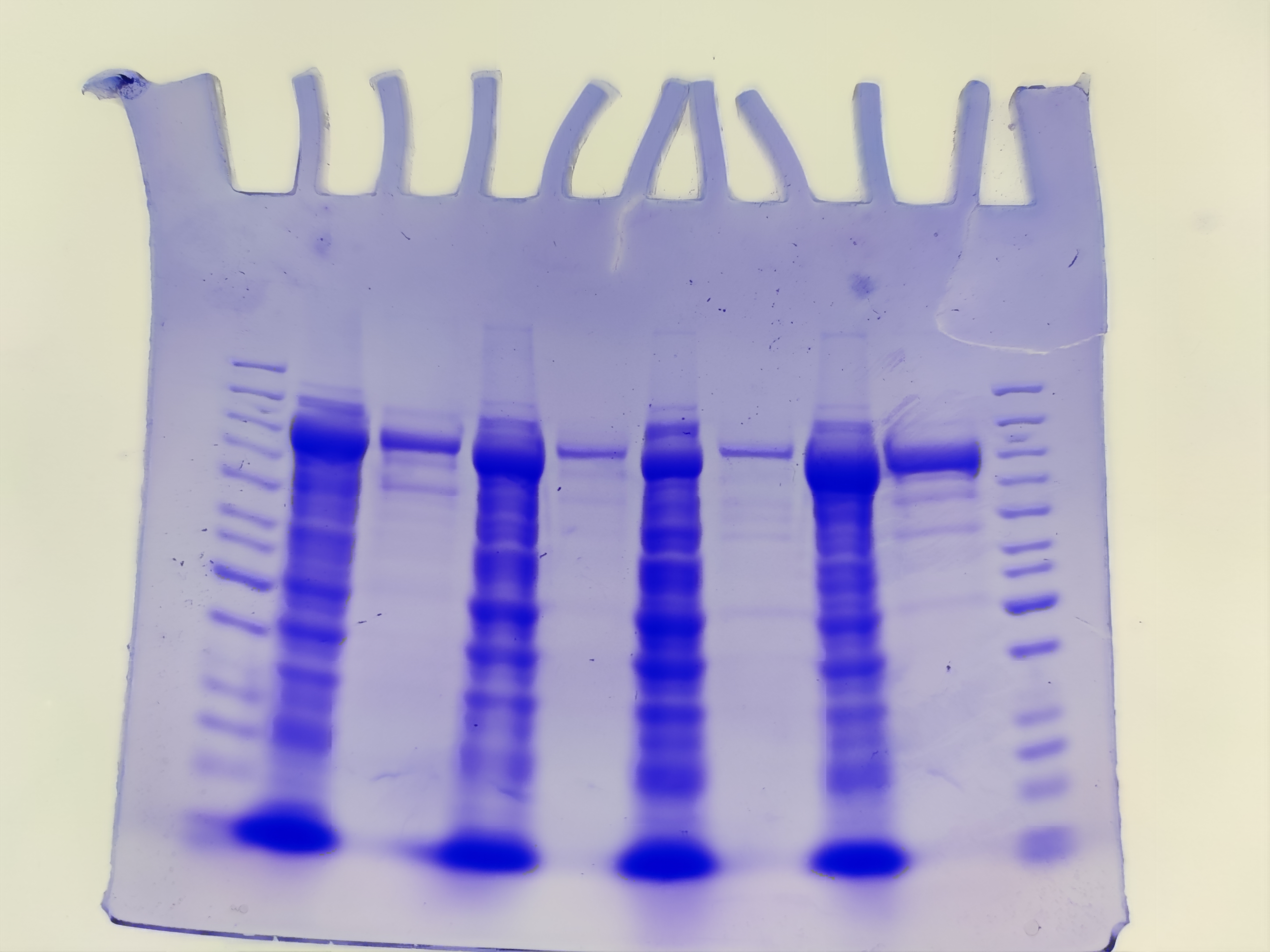

Supplement: Supplementary file 8 [file Image_7.jpeg]

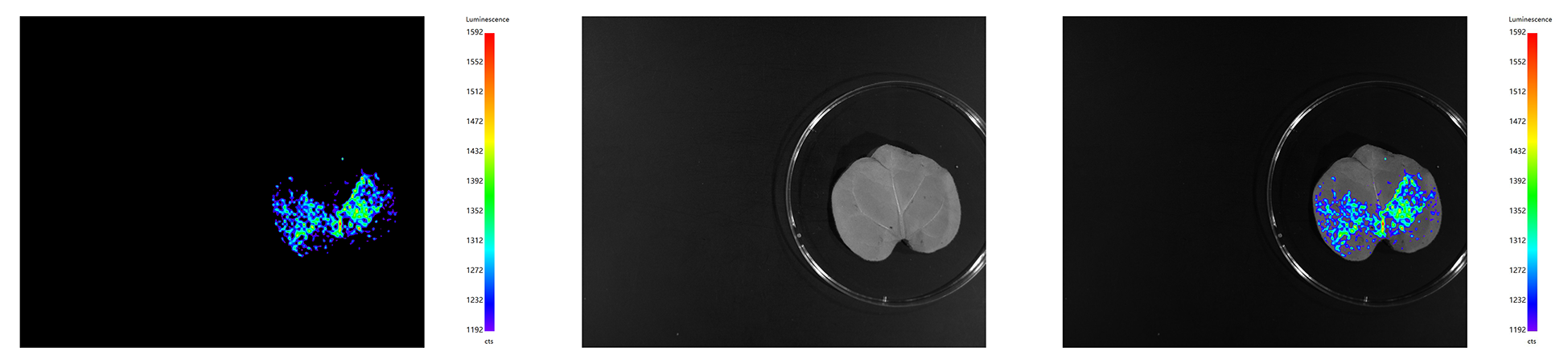

Supplement: Supplementary file 9 [file Image_8.tif]

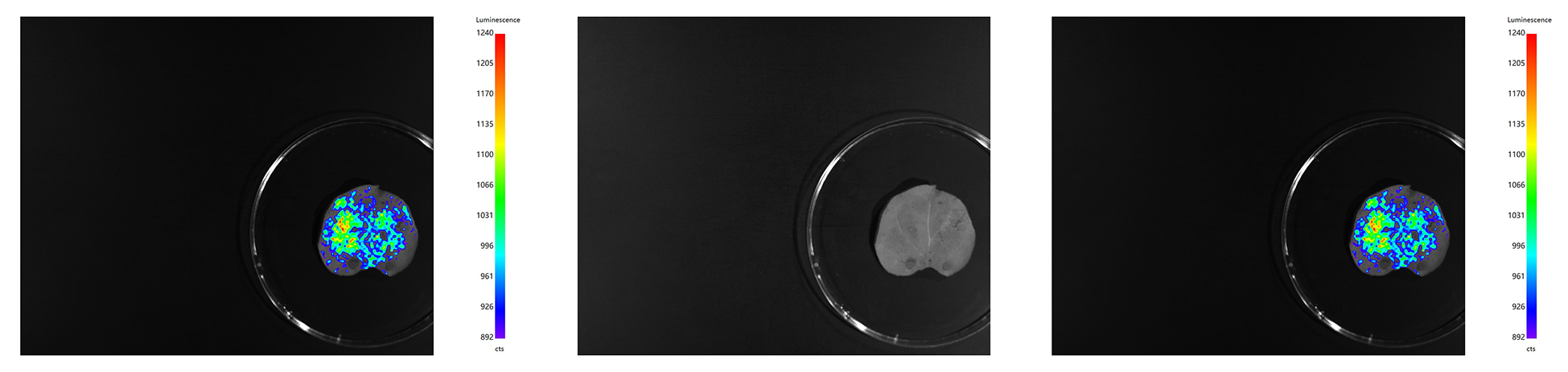

Supplement: Supplementary file 10 [file Image_9.tif]
